# Effects of Reduced Terrestrial LiDAR Point Density on High-Resolution Grain Crop Surface Models in Precision Agriculture

**DOI:** 10.3390/s141224212

**Published:** 2014-12-16

**Authors:** Martin Hämmerle, Bernhard Höfle

**Affiliations:** 1 GIScience Research Group, Institute of Geography, Heidelberg University, Heidelberg 69120, Germany; E-Mail: hoefle@uni-heidelberg.de; 2 Heidelberg Center for the Environment (HCE), Heidelberg University, Heidelberg 69120, Germany

**Keywords:** precision agriculture, 3D geodata, grain crop, LiDAR, resolution, crop surface model, low-cost

## Abstract

3D geodata play an increasingly important role in precision agriculture, e.g., for modeling in-field variations of grain crop features such as height or biomass. A common data capturing method is LiDAR, which often requires expensive equipment and produces large datasets. This study contributes to the improvement of 3D geodata capturing efficiency by assessing the effect of reduced scanning resolution on crop surface models (CSMs). The analysis is based on high-end LiDAR point clouds of grain crop fields of different varieties (rye and wheat) and nitrogen fertilization stages (100%, 50%, 10%). Lower scanning resolutions are simulated by keeping every *n*-th laser beam with increasing step widths *n*. For each iteration step, high-resolution CSMs (0.01 m^2^ cells) are derived and assessed regarding their coverage relative to a seamless CSM derived from the original point cloud, standard deviation of elevation and mean elevation. Reducing the resolution to, e.g., 25% still leads to a coverage of >90% and a mean CSM elevation of >96% of measured crop height. CSM types (maximum elevation or 90th-percentile elevation) react differently to reduced scanning resolutions in different crops (variety, density). The results can help to assess the trade-off between CSM quality and minimum requirements regarding equipment and capturing set-up.

## 1. Introduction

The important role of precision agriculture (PA) for tackling issues like global food supply, adaptation of crops to climatic changes or serving energy needs with renewable biomass is often emphasized [1–5]. Of central importance in PA are geodata of crops because they allow for site-specific management of crop fields [6–10]. Increasingly, three-dimensional geodata (3D geodata) are used in precision agriculture (e.g., [4,11–14]) because they can be examined with spatial (3D) analysis methods. Thus, 3D geodata are the basis for deriving features which cannot be calculated from other data sources like remotely sensed ortho-rectified imagery or classical surveying, e.g., directly captured plant heights [13].

An established method for capturing 3D geodata is LiDAR, which is also increasingly applied in PA-related studies [4,11,13–18]. Advantages of this active remote-sensing method are, e.g., its non-invasive and non-destructive measurements, its capability to capture crops area-wide in high geometric detail, its capability of partly penetrating objects like vegetation, and its independence of lighting conditions [13,15]. LiDAR studies in PA cover a wide range of scanning resolutions and object scales. For example, Paulus *et al.* [17,18] use a high-precision laser scanner coupled with a moveable articulated measuring arm to produce detailed geometric models of single plant organs which are valuable for quantifying growth processes. On a larger scale, crop height models of entire agricultural fields, which can be used, e.g., for biomass estimations are derived from terrestrial laser scanning (TLS) point clouds in [4,14]. In Höfle [13], single maize plant positions and their heights are detected in a 132 m × 6 m field, which is important for growth monitoring and site-specific plant treatment. The study exploits both radiometric and geometric information which were available directly from the TLS point clouds. Eitel *et al.* [16] also use both TLS radiometry and geometry to detect the nitrogen status of wheat for optimizing fertilizer applications.

In the aforementioned studies [4,11,13–17], high quality data are captured with high-end TLS which is not applicable for operational use in PA due to the high cost of equipment, data acquisition and analysis. To overcome these drawbacks, recent studies explore and develop low-cost 3D sensing methods and platforms for different applications and object scales. Chéné *et al.* [19] capture the geometry of single plant components with a low-cost structured-light camera which has been proven to deliver high-quality data [20]. To derive height models for the full extent of grain crop fields, low-cost 2D laser scanners are mounted on a mobile platform in [2,21]. From the captured point clouds, crop height models are derived and successfully used as input for biomass estimations. Similar to PA studies, a need for low-cost 3D sensors is also stated in the neighboring field of forestry. Eitel *et al.* [22], for example, successfully introduce an autonomous low-cost terrestrial laser scanner for monitoring forest growth which allows for continuous scanning of a scene at a fraction of the cost of high-end TLS.

Applying low-cost sensors can lead to datasets with lower resolutions when compared to high-end sensors. In addition, low resolutions can be intentionally chosen to reduce the cost of a scanning campaign [23]. With changing resolutions, the features of point clouds and their derivatives might also change, and influence analysis results. Höfle [13], e.g., finds that by reducing point cloud density, the correctness of single maize plant detection even improves slightly, whereas the completeness drops sharply from about 80% to 12.5% when reducing the point cloud to 10% of its original size (*i.e.*, amount of single measurements).

The effects of lower point cloud densities on derivatives like height-related raster models were mostly analyzed in forestry studies. González-Ferreiro *et al.* [24] state that a reduction of a point cloud to 6% of its original size (16 to 1 point/m^2^ by randomly keeping one point per cell) does not significantly affect the derived stand parameters, which include, e.g., mean height, dominant height, and stand basal area. Similarly, Jakubowski *et al.* [25] find height-related stand parameters react robustly to point cloud thinning until a point density of 1 point/m^2^ is reached. Gobakken and Næsset [26] reduce the density of ALS point clouds of coniferous forest stands by randomly keeping one point per 4, 8, and 16 m^2^ cell (0.25, 0.125, and 0.0625 points/m^2^, respectively). A comparison between original and thinned point clouds shows that especially the maximum canopy height reacts sensitively to reduced point densities with difference values ranging from −0.85 to −2.76 m (at 0.25 and 0.0625 points/m^2^, respectively; [26]).

High-resolution crop surface models (CSM) [27] which represent the upper boundary of a crop field are a crucial input for a wide range of applications in PA. For example, by normalizing a CSM with a digital terrain model representing the elevation of the bare soil, the crop height distribution can be derived, which is used as an input dataset in, for example, grain yield estimations [15], biomass modeling [11,14,21,28], crop nitrogen status [16] and leaf area index distribution [29]. To the knowledge of the authors, no study has investigated the effects of reduced point densities on TLS-based crop surface models for agricultural grain crop fields so far. In this study, we simulate low densities of grain crop field point clouds to analyze the changes in the derived surface models. Other factors affecting data capturing and CSM calculations, and limitations of the sensing method (e.g., laser footprint diameter [30,31], receiver sensitivity [30]) are not examined in this study. Their influence is minimized by selecting datasets which are homogeneous regarding these factors. Based on the results, the trade-off between point cloud density (*i.e.*, data volume, acquisition effort) and surface model quality can be assessed. This study contributes to the improvement of 3D geodata capturing efficiency. It can therefore help to further establish 3D sensors for operational use in precision agriculture by overcoming cost constraints for equipment and data acquisition.

## 2. Study Area and Datasets

**Figure 1.**
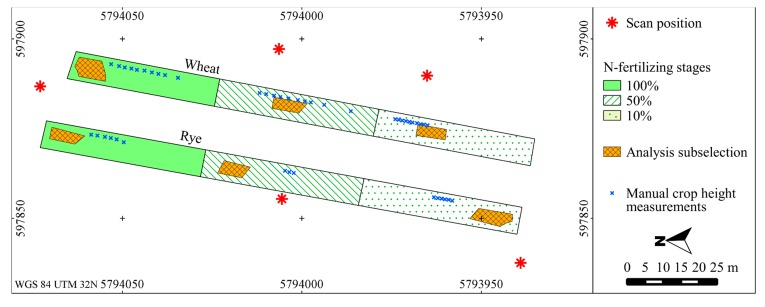
Study area: Scan positions, fertilizing stages and subsampled areas for analysis.

**Figure 2.**
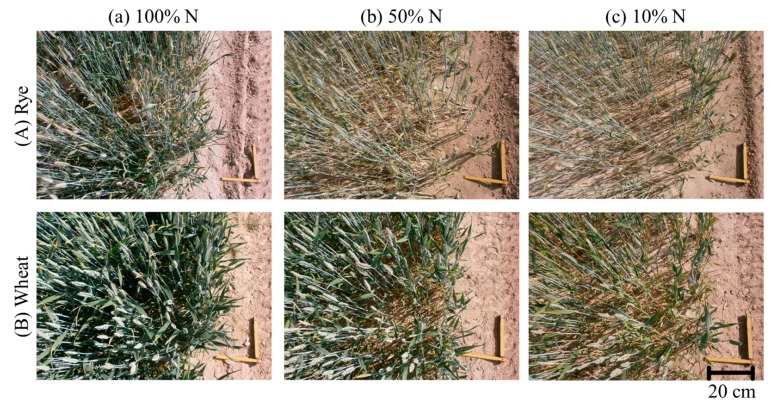
Bird's eye view of the analyzed crop stands. (**a**), (**b**), (**c**) 100%, 50%, 10% nitrogen fertilization, respectively. (**A**) Rye; (**B**) Wheat.

The study area represents a winter rye and a winter wheat field prepared by the Julius-Kühn-Institute for Crop and Soil Science in Brunswick, Germany (52.288° N, 10.435° E). The crops were captured on 6 June 2013 at a growth stage of 71 (rye) and 67 (wheat) according to the BBCH scale [32]. Seed density was 250 grains/m^2^ for rye and 360 grains/m^2^ for wheat. The plain fields of similar growth and soil conditions had a size of 130 m × 6 m and they were subdivided into three parts with different nitrogen fertilizing stages (100%, 50%, and 10%) (Figure 1, Table 1). The different fertilization resulted in different crop stand densities which were visible directly in the fields (Figure 2). To relate CSM elevation changes to actual crop heights, the maximum height of single plants was measured at multiple locations in each fertilizing stage and the mean value of the measurements was derived (Figure 1, Table 1).

The applied time-of-flight VZ-400 laser scanner (Riegl, Horn, Austria) emits near-infrared beams of 1550 nm wavelength and 0.35 mrad divergence (3.5 mm diameter at 10 m distance). Its range accuracy is 5 mm (one sigma) at 100 m distance according to the data sheet [33]. The fields were scanned with the scanner mounted about 4 m above ground on an elevated platform. High-resolution point clouds for the experiments were acquired with a point spacing of 5 mm at 10 m distance. Additionally, low-resolution scans were captured from the same scan positions with a point spacing of 17 mm at 10 m distance. They are the basis for direct comparisons to the simulated low-resolution scans generated in this study.

To assess the effects of point density reduction on crop surface models in areas of different crop stand densities, point cloud subsamples containing single and first echoes were extracted from each fertilizing stage. To achieve similar scanning geometries for the subsamples, a range filter from 12 m to 19 m was applied to the point cloud of the scan position closest to the respective fertilizing stage plot (Figure 1), which is the range available in all fertilizing stages. Within this range, factors which are not in the focus of this study but which also influence the derivation of CSMs (e.g., laser footprint diameter [30,31]) are considered to be constant. The occurrence of missing recordings due to low echo energy at the maximum scanning range [30] is also assumed to be minimized by applying the range filter as the selected areas are close to the scanner and well within the maximum scanning range of up to 600 m [33]. The number of laser points that penetrate the crop stand horizontally via the field border was reduced by including a distance of minimum 1 m to the crop field boundary for subsampling. Vertical outliers were removed manually using the Riegl RiSCAN PRO software. The six resulting point clouds (Table 2) were exported into ASCII files which contain the XYZ coordinates for the analyses.

## 3. Methods

**Figure 3.**
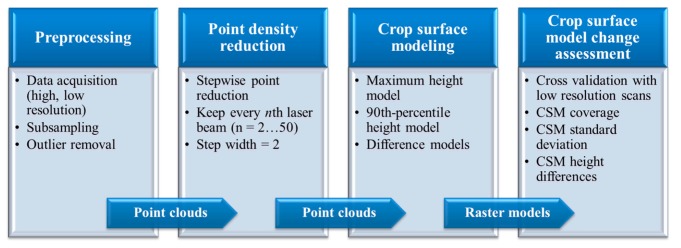
Overview workflow of the study design.

The main steps conducted in this study are summarized in Figure 3. Data preparation and preprocessing were described in Section 2. In Section 3.1., the method for reducing the point density and the resulting datasets are introduced, followed by details on the crop surface modeling and crop surface model change assessment in Section 3.2.

### 3.1. Point Density Reduction

**Figure 4.**
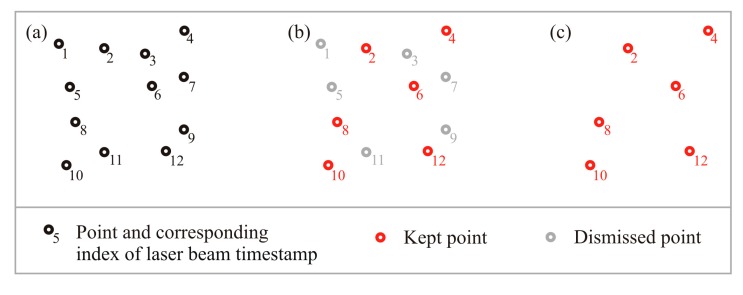
Stepwise point density reduction. (**a**) Original point cloud with index of laser beam timestamp; (**b**) Stepwise point selection with step width 2; (**c**) Resulting reduced point cloud.

To simulate lower scanning resolutions, the number of points is reduced in each of the six sample point clouds by selecting the point of every *n*th laser beam based on the timestamp of beam emission, with *n* being a multiple of two (Figure 4a). The approach simulates a scanning set-up with lower scanning frequencies of a static terrestrial scanner as introduced, e.g., in [22]. The steps applied in this study range from 2 to 50 so that 25 reduced point clouds were generated from the original dataset. The final step width of 50 is chosen to reach the point cloud reduction which corresponds to the data of the low-resolution scans. The stepwise reduction approach is also used, e.g., in [34,35].

### 3.2. Crop Surface Modeling and Surface Model Change Assessment

In order to assess the effect of point cloud thinning on crop surface models, two CSM types are derived and analyzed: (1) a raster Z_max_ with the maximum LiDAR point elevation value assigned to the respective cell and (2) a raster Z_p90_ with cells containing the elevation of the 90th percentile. Z_max_ is chosen to analyze a simple and straight forward canopy surface model which is, for example, appropriate for on-line processing. With Z_p90_, a model is analyzed which is more robust against changes in point density than Z_max_ [24–26]. The CSMs are calculated with a cell size of 0.1 m × 0.1 m which is the cell size that results in a seamless CSM derived from the high-resolution (5 mm at 10 m distance) point clouds (CSM_ref_). In the CSMs which are derived from thinned point clouds, raster cells with no value can occur because the respective cells are void of laser points. These raster gaps are included and evaluated in the subsequent analyses and, thus, are intentionally not interpolated as it was done in other studies, e.g., [14,27].

The assessment of the CSMs derived from the thinned point clouds (CSM_red_) is based on the comparison between the CSM_ref_ and CSM_red_ raster datasets. Thus, this study follows an intrinsic approach [36] which allows for a relative assessment based on features derived from the datasets themselves, as applied, e.g., in [37–39] who use their maximum quality datasets as a basis for reference models and model change assessments. The overlap of CSM_ref_ and the respective CSM_red_ is guaranteed by a fixed coordinate of the raster origin. All calculations were conducted in the software Orientation and Processing of Airborne Laser Scanning Data (OPALS) [40].

In order to validate the CSM_red_, they are compared to their counterparts derived from the low-resolution scan datasets (CSMs_lowres_). Number of points, coverage (*i.e.*, number of raster cells with elevation values relative to the seamless CSM_ref_), standard deviation of CSM elevation values, and the mean absolute elevation difference to the CSM_ref_ are compared. CSM_red_ and CSMs_lowres_ are connected via the absolute number of points in the respective point clouds: In the 10%-fertilized rye dataset, for example, the reduction step width 48, with 3221 points, is closest to the low-resolution scan, with 3182 points, and is therefore used for the comparison.

To analyze the CSM_red_ development over the whole range of reduction steps, coverage, standard deviation, and the mean absolute difference values CSM_ref_ − CSM_red_ are calculated for each reduced point cloud (Table 3). The three parameters are chosen to represent core features of the CSMs: The coverage describes the ratio of cells relative to the CSM_ref_ which contain at least one laser point and thus an elevation value. On the basis of the coverage, the required scanning resolution for deriving seamless CSMs or CSMs that contain a given percentage of empty cells can be assessed. The standard deviation of CSM elevation values (SD) represents the vertical heterogeneity of the crop surface. It influences, e.g., the application of fungicides, which depends on the crop surface area [29]. If the SD changes with reduced scanning resolutions, an adjustment of the herbicide quantity would be necessary. The mean absolute differences CSM_ref_ − CSM_red_ represent the decreasing probability of capturing the highest points in a crop canopy with decreasing scanning resolution. All applications that are based on CSMs or their derivatives are, thus, affected by CSM_ref_ − CSM_red_ differences, which implies the need for corrected CSM elevation values when lower scanning resolutions are used for capturing crop fields.

## 4. Results

The results of stepwise thinning of the high-resolution datasets are described in Section 4.1. Section 4.2 contains the comparison between the stepwise-thinned point clouds and their low-resolution counterparts as well as the description of the changes of the CSM parameters coverage, standard deviation, and the mean elevation difference in absolute values and percentage of crop height.

### 4.1. Effects of Point Density Reduction

**Figure 5.**
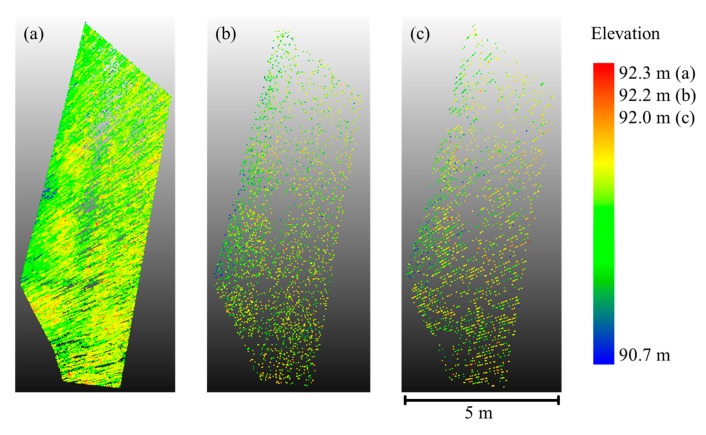
Exemplary results for point density reduction. Bird's eye view on the point cloud of 10%-fertilized rye. (**a**) Original point cloud colored according to point elevation; (**b**) Result of point cloud thinning with step width *n* = 48; (**c**) Point cloud of low-resolution scan with a number of points similar to (b). (b) and (c) are also colored according to point elevation.

Figure 5 shows an exemplary result of the stepwise point cloud reduction. The stepwise reduction method follows closely a 1/*n* relationship with *n* being the step width (*i.e.*, every *n*-th point of the full dataset is selected). The number of points in the point cloud shown in Figure 5b is 2.09% of the original dataset, corresponding well to the theoretical percentage of 2.08% derived from step width 48. The horizontal extent and high point density clusters are preserved by the method whereas areas with a low number of points tend to become void of points. The point cloud which was extracted from the coarse scan (Figure 5c) exhibits a similar point distribution but with a more emphasized distinction between high and low point density areas. The vertical extent of the thinned (Figure 5b) and low-resolution (Figure 5c) point cloud is 0.1 m and 0.3 m smaller, respectively, which reflects the lower probability of capturing single protruding stalks when applying lower resolutions.

### 4.2. Crop Surface Modeling and Surface Model Change Assessment

To compare the point clouds produced by low-resolution scans with the corresponding point cloud resulting from the stepwise reduction process, descriptive features are summarized in Table 4. Additionally, coverage, standard deviation, and the mean value of the difference to the CSM_ref_ are listed for the CSMs_lowres_ and CSMs_red_.

Regarding the point density, the coarse scans and the reduced point clouds have a similar number of points per cell of 0.4 points/0.01 m^2^ in average. On the other hand, the coverage of the derived CSMs is lower for the CSMs_lowres,_ especially in case of the Z_max_ CSM with a mean coverage of 44.6% for the CSMs_lowres_ and 51.2% for the CSMs_red_. For the Z_p90_ surface models, the coverage is similar with 19.6% (CSM_lowres_) and 18.2% (CSM_red_). Also the standard deviations of the CSM elevation values and the mean difference to the CSM_ref_ correspond well with the difference values being at least one order of magnitude smaller.

**Figure 6.**
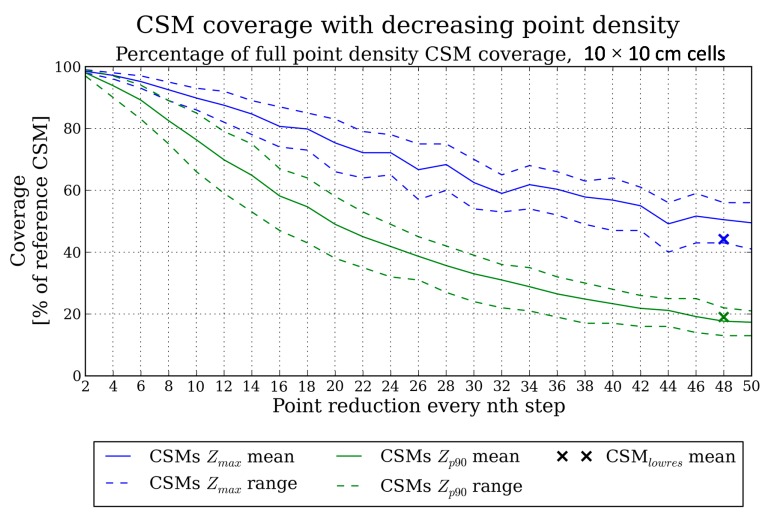
Development of mean CSM_red_ coverage with stepwise reduced point cloud size. Solid line: Mean coverage value of all analyzed crop plots, dashed line: range of coverage values. Blue: Z_max_ CSM, green: Z_p90_ CSM. Cross markers: Mean CSM_lowres_ values.

**Figure 7.**
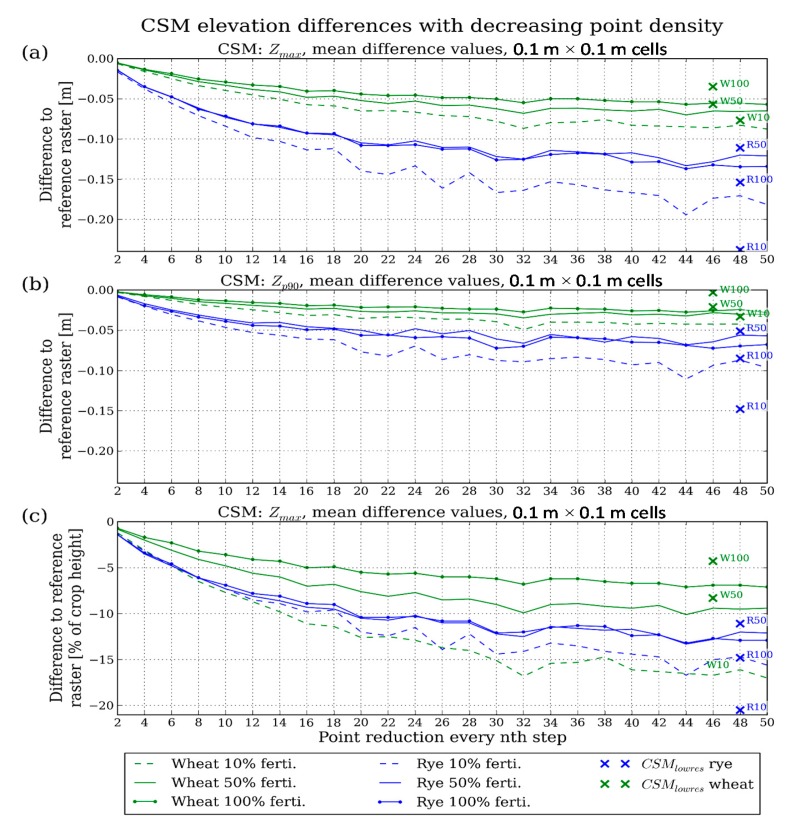
Development of mean CSM_ref_ − CSM_red_ elevation difference values with stepwise reduced point cloud size. (**a**), (**b**) Absolute values for Z_max_ and Z_p90_ CSMs, respectively; (**c**) Values for Z_max_ in percent of crop height (manual field measurements). Blue: rye plots, green: wheat plots. Solid line with markers: 100% fertilizing, solid line: 50% fertilizing, dashed line: 10% fertilizing. Crosses: CSM_lowres_ values at the corresponding reduction step width. Cross indices: ‘R’ = rye, ‘W’ = wheat, number = fertilizing stage.

The following diagrams (Figures 6 and 7) describe the CSM_red_ changes with decreasing point density by showing the development of the parameters chosen for characterizing the CSMs_red_ over the whole range of point cloud thinning.

The coverage relative to the CSM_ref_ is summarized in Figure 6. To relate the calculations to the CSMs_lowres_, the mean coverage values of Table 4 are shown as crosses at step width 48. The most prominent feature of the parameter development in Figure 6 is the general decrease of coverage values. For example, a reduction to 25% of the original point cloud size (step width 4) still leads to a high coverage of 97.2% and 93.8% for Z_max_ and Z_p90_ models, respectively. However, starting from the first reduction steps, a divergence between the Z_max_ and Z_p90_ CSM_red_ values can be seen. This can be attributed to the exclusion of cells which contain only one point in the Z_p90_ CSM and which are not used for the derivation of elevation percentile values. It occurs mainly in the first half of the reduction steps until about step width 26, finally leading to a coverage of 49.5% for the Z_max_ CSM_red_ and 17.3% for the Z_p90_ CSM_red_ at step width 50.

When differentiating between crops and fertilizing stages, the CSMs_red_ show a similar behavior in both the Z_max_ and Z_p90_ models. The lowest loss of coverage is calculated for the 10%-fertilized wheat plot. The descending order of the other datasets is 50%, 100%-fertilized wheat and 10%, 100%, 50%-fertilized rye. Therefore, the wheat plot CSM coverage is less sensitive against point cloud reduction. The least fertilized plot shows the lowest coverage loss when comparing the fertilizing stages for one crop variety. This can be explained by the more regular penetration of laser beams in the less dense stands, so that the points available for CSM raster derivation are distributed more homogeneously within the CSM extent and, thus, fewer cells void of points occur.

Finally regarding the CSMs_red_ and CSMs_lowres_ coverage values, the Z_p90_ CSM derived from reduced point clouds shows practically no difference. Contrary, the coverage of the Z_max_ CSM_red_ is too high when compared to the CSM_lowres_ which is due to the more homogeneous distribution of points in the thinned point clouds (Figure 5). The CSM_red_ coverage should thus be considered too optimistic in case of the Z_max_ CSMs.

The standard deviations show no distinct change all over the different reduction steps. For both the Z_max_ and Z_p90_ CSMs_red_, a slight increase of values occurs which follows a linear function with 0.0012 (Z_max_) and 0.0006 (Z_p90_) slopes. At the same time, the mean elevation values of the CSMs_red_ exhibit slight decreases with slopes of −0.0028 (Z_max_) and −0.0008 (Z_p90_). In addition to the point density reduction, the observed standard deviation changes are also influenced by changing mean values. However, the magnitude of change in the standard deviation is marginal, so that the standard deviation of CSM_red_ elevation values is considered constant over the whole range of reduction steps. Models and applications that use the standard deviation of CSM elevation as input can therefore be regarded as robust against reducing the scanning resolution.

A further important feature examined in this study is the CSM elevation change. Figure 7a,b shows the mean absolute elevation difference CSM_red_ – CSM_ref_ for Z_max_ and Z_p90_ surface models. Similar to the development of coverage (Figure 6), the CSM_red_ mean elevations decrease with increasing point reduction, following logarithmic functions similar to the results in [25,41] given for forest stands. When comparing the Z_max_ (Figure 7a) and Z_p90_ (Figure 7b) CSMs, the Z_max_ models clearly react stronger to the point reduction with the final values at step width 50 being on average two times higher than the respective Z_p90_ values. Corresponding to the findings in forestry studies [24–26], the more robust Z_p90_ CSM can therefore also be considered advantageous for applications in PA.

The CSM elevation development also differs between the two crop varieties, with wheat reacting less sensitively to point reduction than rye. This is the case for the Z_max_ and the Z_p90_ CSMs, but more emphasized for the Z_max_ models. In both crop varieties, the two higher fertilized plots show a similar development of mean CSM_red_ elevation values whereas the 10% fertilizing stages show a stronger decrease, and thus diverge from the 100% and 50% fertilizing stages.

The comparison between CSM_red_ and CSM_lowres_ indicates that the CSMs_red_ tend to overestimate the reduction of CSM elevation in the case of the wheat plots in both the Z_max_ and the Z_p90_ models (Figure 7a,b; Table 4). Taking the example of the 100%-fertilized wheat, the Z_p90_ model derived from the thinned point cloud shows an elevation difference to the CSM_ref_ of 0.026 m, but the Z_p90_ model which is based on the low-resolution scan has an even lower difference of 0.003 m so that almost no reduction of CSM elevation over the whole range of simulated resolutions can be expected when conducting real scans. In contrast, in the case of the rye plots, the CSM_lowres_ values cover a wider range, leading to a slight overestimation of CSM elevation difference in the 50%-fertilized rye plot and a strong underestimation in case of the 10%-fertilized plot (Figure 7a,b).

Overall, the 100%-fertilized wheat, which can be considered the plot with the highest crop density due to seed density and maximum fertilization, is least affected by point cloud reduction. At the final reduction stage (step width 50, 2% of the original point cloud size), the CSM_red_ is in average 5.7 cm and 2.8 cm lower in the Z_max_ and Z_p90_ CSM, respectively. In contrast, the 10%-fertilized rye plot shows the largest CSM elevation decrease with 18.1 cm (Z_max_) and 9.6 cm (Z_p90_).

In addition to the absolute CSM difference values, the mean elevation difference relative to crop height is of interest. In Figure 7c, the difference values of the Z_max_ CSMs are therefore related to the manual crop height measurements (Table 1) and given in percent of crop height. A different pattern can be seen when compared to Figure 7a. The rye plot CSMs do not strictly show the highest elevation reduction values any more as the 10%-fertilized wheat has the highest percentage of CSM elevation change. Furthermore, the rye plots show a similar development over the range of reduction steps and diverge less than in Figure 7a. However, the rye plots still react more sensitively to point reduction compared to 100% and 50%-fertilized wheat.

Overall, the 10%-fertilized wheat plot shows the highest percentage of elevation differences with up to 17.0% of the crop height at reduction step 50, although the comparison to the CSM_lowres_ value indicates an overestimation of the difference. In addition, the divergence from its higher fertilized counterparts is more emphasized in comparison to Figure 7a. Similar to Figure 7a, the robustness of the 100%-fertilized wheat plot data against point reduction stands out. At a reduction to 6.25% of the original point cloud size (step width 16), the mean CSM_red_ elevation is only 5% lower than the measured crop height. In contrast, 10%-fertilized wheat shows a decrease of 11% at the same point reduction level.

## 5. Discussion

A prominent feature when comparing the CSM elevation changes in Figure 7a,b is the high sensitivity of the Z_max_ CSM, which is consistent with the findings in [24–26]. Thus, depending on the application in PA, the appropriate model type must be chosen. If, for example, a CSM were used to adjust the height of an agricultural processing tool to the crop surface, the Z_max_ CSM would better describe the maximum surface elevation, and a high scanning resolution would be required. In contrast, for empirical models of, e.g., biomass as used in [11] for optimizing the speed of a combine harvester, a Z_p90_ CSM can be used as input, thus reducing the required scanning resolution.

In addition, the different crop varieties and densities examined in this study lead to different sensitivities to point density reduction. The 100%-fertilized wheat, for example, results in the lowest percentage of elevation loss with decreasing point density due to a very dense and plain canopy (indicated by low CSM standard deviations in Table 4), which intercepts most of the laser beams. The resulting point cloud resembles a plain that is still captured well by low-resolution scans. The described behavior may seem trivial, because by applying a lower scanning resolution, the probability of capturing the highest point of a plant obviously decreases. However, there are crucial implications for precision agriculture: To decide on the applied scanning resolution or the scanning device, there needs to be *a priori* knowledge about the crop stand features. If, for example, a mean Z_max_ CSM elevation loss of 7% is feasible, a wheat field with a crop density and height distribution similar to the 100%-fertilized field used in this study can be scanned with 1/50th of the resolution applied here, meaning that instead of 0.005 m point spacing at 10 m distance, 0.25 m would be sufficient (Figure 7c). But when working on a field similar to the 10%-fertilized wheat of this study, a point spacing of 0.04 m would be required to reach the same CSM quality.

Similarly, for example, when correction factors for biomass or leaf area index models have to be provided, generic values cannot be applied. The models must be adjusted to the crop features, as stated also in [42] for forestry applications. To find correction factors for homogeneous parts of a crop field, representative areas can be captured with both LiDAR and manual reference measurements.

The crop stand features also influence the robustness of CSM coverage: In sparse and homogeneous crops like the 10%-fertilized wheat used in this study, the penetration rate of laser beams is higher. By reducing the number of points of the 10%-fertilized wheat point cloud to 1/22 of its original size, a Z_max_ CSM coverage of 80% could still be achieved. To capture a crop field similar to the 10%-fertilized wheat used in this study, lower resolutions still provide enough measurements to derive a CSM with high coverage. In denser or more heterogeneous fields, on the other hand, more laser beams are intercepted in the canopy or single dense areas as caused, e.g., by dense tussocks, so that the crop stand is not homogeneously covered with measurements. Subsequently, compared to the example of 10%-fertilized wheat, the 50%-fertilized rye only shows a coverage of 64%, so that in comparable crop stands, higher scanning resolutions would be necessary for deriving seamless CSMs.

The two CSM types (Z_max_, Z_p90_) show different sensitivities of coverage against point density reduction, with the Z_p90_ model being more sensitive (Figure 6). Thus, taking into account the higher sensitivity of Z_p90_ CSMs, there is a trade-off between coverage and elevation accuracy. However, it must be noted that this applies for the 0.1 m × 0.1 m raster as used in this study, which was chosen to achieve a seamless high-resolution CSM in accordance to other studies using the same [13], or even higher [11,14], resolutions.

Finally, issues regarding the used datasets and methods are addressed. First, it should be kept in mind that the point cloud reduction by selecting points of every *n*th laser beam simulates low-resolution scans. The high-resolution point clouds used as a basis for the reduction contain measurements that were captured due to a higher penetration rate compared to a low-resolution scan. By starting the simulation of a low-resolution scan from a high-resolution dataset, points will be included in the selection of every *n*th laser beam that would not have been captured in case of a real low-resolution scan at all. This can be seen in Figure 5 as well as in the deviations between CSMs_red_ and CSMs_lowres_ in Figures 6 and 7. However, the comparison between the CSMs_red_ and CSMs_lowres_ confirms, rather than contradicts, the presented results so that the simulated low-resolution datasets are considered valid representatives of low-resolution scans.

Second, to achieve datasets of comparable scanning conditions, plots within the same scanning range were selected, resulting in low incidence angles around 75° against nadir. Higher incidence angles are normally preferred to avoid shadowing effects in canopies [11,43,44] as visible in Figure 5a. However, as the object examined in this study was the crop surface and not the volume or the terrain, the low penetration rate that can be expected from the low incidence angle was not considered an obstacle for the analyses.

Third, no interpolation method was applied to fill gaps in the derived CSMs due to cells void of laser points. The presented results, especially regarding the coverage values, can therefore be used as guidelines to assess the percentage of CSM cells that would have to be filled by interpolation methods, should a seamless CSM need to be provided as, e.g., in [14].

## 6. Conclusions and Outlook

To analyze the effect of point density on high-resolution grain crop surface models, high-end TLS point clouds with a resolution of 5 mm at 10 m distance were thinned by stepwise reduction of the number of points. By comparing the derived CSMs it was found that when reducing the point cloud to, e.g., 25% of the number of points in the high-end dataset, the CSM coverage (*i.e.*, cells that contain at least one point) is still >90% (Figure 6) and the average CSM elevation difference is <4% of crop height (Figure 7c). However, coverage and elevation sensitivity strongly depend on CSM type (e.g., Z_max_ or Z_p90_) and crop stand features (e.g., surface heterogeneity, crop density). The coverage of Z_max_ CSMs is more robust against point cloud thinning compared to Z_p90_ CSMs, but the Z_max_ CSM elevation reacts more sensitively. Additionally, the CSM coverage of the sparse crop stands was less sensitive compared to the dense stands. In contrast, the CSM elevation of the dense crop stands results in a higher robustness against reducing the number of points. Thus, depending on the application, a trade-off between coverage and robust elevation modeling has to be taken into account by choosing the appropriate CSM type and scanning resolution. Furthermore, when using CSMs as input for, e.g., biomass or leaf area index models, correction factors have to be derived from on-site measurements to cover the crop height and density heterogeneities within a field.

The applied reduction method approximates datasets produced by scanning devices with lower performance and cost, or scan settings which were chosen to capture smaller data volumes. A comparison between high-end datasets and data produced by real low-cost TLS as used, e.g., in [22,45,46] would be a further important step towards optimized 3D or, by adding the time dimension, 4D analysis of grain crop fields. In addition, other scanning set ups like 2D laser scanners mounted on mobile platforms [11,21,43] or unmanned aerial vehicle-borne laser scanning (ULS) [15,45,47–50] should be investigated regarding the minimum requirements for deriving models that are of value for PA applications. Homogeneous footprint sizes, nadir perspectives, and scanning ranges can be achieved by mounting multiple 2D scanners on a boom spanning the working width of an agricultural machine. The mentioned scanning set ups can provide advantageous scanning geometries for penetrating a crop stand to the ground and for avoiding disadvantages like, e.g., angle-dependent errors as examined in [51]. A combination of multiple low-cost sensors scanning the area closely in front of an agricultural machine over the whole working width could thus be used for deriving, e.g., canopy height or biomass models on-site and on-line, providing information for site-specific crop treatment and growth monitoring in real time. Scanning devices operating with larger laser footprint diameters compared to the 3.5 mm at 10 m distance applied in this study were used in [4,11,43], with up to 140 mm at 10 m distance in [4]. Examining the optimal footprint diameter for capturing objects like grain crop fields similar to the forestry studies [30,31] would be beneficial for improving the efficiency of capturing 3D geodata in PA. Furthermore, from point clouds captured by a device providing the full waveform of large footprints, the vertical structure of crop stands could be derived as shown for a forested area in [52]. Another promising method for the 3D reconstruction of crop fields is based on imagery captured from cameras mounted on unmanned aerial vehicles (UAV). Using the structure from motion-approach (SfM) to derive 3D geodata from UAV-based photographs, the studies [12,53] show a high potential of UAV use in precision agriculture. The transfer of the findings presented in this study to other sources of point clouds of agricultural objects, e.g., the SfM method, should be investigated to find new, efficient and complementary sources for 3D geodata important for precision agriculture.

## Figures and Tables

**Table 1.** Fertilizer application and manual plant height measurements in the analyzed crop stands. The number after the crop name indicates the nitrogen fertilizing stage in percent.

**Crop**	**Fertilization [kg/ha]**	**Manual Measurements of Maximum Plant Height**	

		**Number of Measurements**	**Mean [m]**
Rye 10	1 × 10	6	1.16
Rye 50	2 × 25	3	1.00
Rye 100	2 × 50	6	1.04
Wheat 10	1 × 16	10	0.52
Wheat 50	2 × 40	10	0.69
Wheat 100	2 × 80	10	0.81

**Table 2.** Features of the high-resolution point clouds. The number after the crop name indicates the nitrogen fertilizing stage in percent.

**Crop**	**Extent [m × m]**	**Number of Points**	**Point Density [Points/0.01 m^2^]**		

			**Mean**	**Maximum**	**Standard Deviation**
Rye 10	4 × 11	153,940	18.6	222	30.4
Rye 50	3.5 × 8	74,493	15.0	142	21.0
Rye 100	3 × 8	80,969	13.0	170	21.5
Wheat 10	3 × 8	100,169	16.5	148	24.2
Wheat 50	3 × 9	99,077	23.8	122	23.6
Wheat 100	5 × 9	154,057	23.4	224	27.3

**Table 3.** Crop surface model parameters examined in this study.

**Parameter**	**Description**	**Represented Feature**
Coverage	Percentage of cells with elevation values relative to the seamless CSM_ref_	Horizontal distribution of points
Standard deviation	Standard deviation of the elevation values of the derived CSMs	Vertical distribution of points and elevation values in the derived CSMs
Mean difference to CSM_ref_	Mean elevation difference CSM_ref_ − CSM_red_ or CSM_ref_ − CSM_lowres_	Measure of CSM height underestimation due to reduced data resolution

**Table 4.** Point cloud and CSM features of the low-resolution scans (‘Lowres’) and their counterparts derived from stepwise point cloud thinning (‘Step *n*’).

**Crop**	**Point Cloud**	**No. of Points (abs.)**	**No. of Points (Mean)**	**CSM Type**	**Coverage**		**Standard Deviation**		**CSM_ref_ − CSM_red/lowres_ (Mean)**	
			
			**[Points/0.01 m**^2^**]**		**[%]**	**diff.**	**[m]**	**diff.**	**[m]**	**diff.**
R10	Lowres	3182	0.4	Z_max_	44.2		0.214		0.24	
				Z_p90_	21.8		0.191		0.15	

	Step 48	3221	0.4	Z_max_	53.7	−9.5	0.225	−0.011	0.17	0.10
				Z_p90_	22.4	−0.6	0.179	0.012	0.09	0.06

R50	Lowres	1585	0.3	Z_max_	37.7		0.124		0.11	
				Z_p90_	15.7		0.110		0.05	

	Step 48	1546	0.3	Z_max_	43.7	−6.0	0.122	0.002	0.12	−0.01
				Z_p90_	13.3	2.4	0.097	0.013	0.06	−0.01

R100	Lowres	1670	0.3	Z_max_	36.8		0.122		0.15	
				Z_p90_	16.2		0.109		0.08	

	Step 48	1680	0.3	Z_max_	45.6	−8.8	0.125	−0.003	0.13	0.02
				Z_p90_	14.9	1.3	0.100	0.009	0.07	0.01

W10	Lowres	2110	0.4	Z_max_	51.1		0.094		0.08	
				Z_p90_	22.9		0.078		0.03	

	Step 48	2076	0.4	Z_max_	56.6	−5.5	0.087	0.007	0.08	0.00
				Z_p90_	21.6	1.3	0.067	0.011	0.04	−0.01

W50	Lowres	2123	0.5	Z_max_	49.0		0.062		0.06	
				Z_p90_	20.4		0.050		0.02	

	Step 46	2,127	0.5	Z_max_	53.7	−4.7	0.060	0.002	0.07	−0.01
				Z_p90_	18.4	2.0	0.046	0.004	0.03	−0.01

W100	Lowres	3396	0.5	Z_max_	48.8		0.049		0.03	
				Z_p90_	20.7		0.051		0.00	

	Step 46	3326	0.5	Z_max_	53.6	−4.8	0.053	−0.004	0.06	−0.03
				Z_p90_	18.7	2.0	0.046	0.005	0.03	0.00

**Average**	**Lowres**	**--**	**0.4**	**Z_max_**	**44.6**		**0.111**		**0.11**	
				**Z_p90_**	**19.6**		**0.098**		**0.04**	

	**Step**	**--**	**0.4**	**Z_max_**	**51.2**	**−6.6**	**0.112**	**−0.001**	**0.11**	**0.00**
				**Z_p90_**	**18.2**	**1.4**	**0.089**	**0.009**	**0.05**	**−0.01**
